# Hyperuricemia Is Independently Associated With Hyperlipidemia in a Large Population‐Based Study

**DOI:** 10.1002/jcla.70131

**Published:** 2025-11-25

**Authors:** Ta‐Jen Lin, Jia‐In Lee, Szu‐Chia Chen, Shu‐Pin Huang, Jiun‐Hung Geng

**Affiliations:** ^1^ Department of Post‐Baccalaureate Medicine, College of Medicine Kaohsiung Medical University Kaohsiung Taiwan; ^2^ Department of Psychiatry, Kaohsiung Medical University Hospital Kaohsiung Medical University Kaohsiung Taiwan; ^3^ Department of Internal Medicine, Kaohsiung Municipal Siaogang Hospital Kaohsiung Medical University Kaohsiung Taiwan; ^4^ Department of Internal Medicine, Division of Nephrology Kaohsiung Medical University Hospital, Kaohsiung Medical University Kaohsiung Taiwan; ^5^ Faculty of Medicine, College of Medicine Kaohsiung Medical University Kaohsiung Taiwan; ^6^ Research Center for Environmental Medicine Kaohsiung Medical University Kaohsiung Taiwan; ^7^ Graduate Institute of Clinical Medicine, College of Medicine Kaohsiung Medical University Kaohsiung Taiwan; ^8^ Department of Urology Kaohsiung Medical University Hospital, Kaohsiung Medical University Kaohsiung Taiwan; ^9^ Department of Urology, School of Medicine, College of Medicine Kaohsiung Medical University Kaohsiung Taiwan; ^10^ Department of Urology Kaohsiung Municipal Siaogang Hospital Kaohsiung Taiwan

## Abstract

**Background:**

Hyperlipidemia is a significant risk factor for cardiovascular diseases. Hyperuricemia has also been linked to adverse cardiometabolic outcomes, including dyslipidemia. The relationship between hyperuricemia and hyperlipidemia is of particular concern due to their combined impact on cardiovascular risk.

**Methods:**

This study analyzed data from 119,037 participants in the Taiwan Biobank to explore the association between hyperuricemia and hyperlipidemia. The study compared baseline characteristics and metabolic profiles between participants with and without hyperuricemia. Univariate and multivariate logistic regression analyses were conducted to identify factors associated with hyperlipidemia, focusing on hyperuricemia as an independent variable.

**Results:**

The participants with hyperuricemia had a more adverse cardiometabolic profile, including a higher body mass index (BMI), a higher prevalence of smoking and alcohol consumption, hypertension, and diabetes, and a worse lipid profile. Univariate analysis showed significant associations between hyperlipidemia and age, BMI, smoking history, alcohol consumption, hypertension, diabetes, and hyperuricemia (OR: 1.706, 95% CI: 1.625–1.791, *p* < 0.001). In multivariate analysis, hyperuricemia remained an independent predictor of hyperlipidemia (OR: 1.102, 95% CI: 1.041–1.165, *p* < 0.001), along with age, BMI, smoking, alcohol consumption, hypertension, diabetes, and a lower estimated glomerular filtration rate.

**Conclusion:**

Hyperuricemia is independently associated with hyperlipidemia, underscoring its role in the complex interplay of metabolic factors contributing to cardiovascular disease risk. Our findings highlight the importance of comprehensive risk factor management incorporating serum uric acid to mitigate the impact of hyperlipidemia and associated cardiovascular conditions.

## Graphical abstract

This study investigated the association between hyperuricemia and hyperlipidemia using data from 119,037 participants in the Taiwan Biobank. The findings revealed that hyperuricemia is independently associated with an increased risk of hyperlipidemia, emphasizing its role in cardiovascular risk and the need for integrated metabolic risk management.

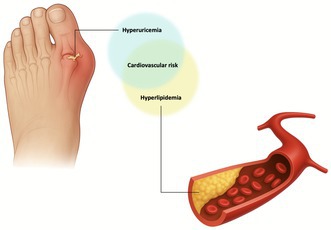

## 1 Introduction

Hyperlipidemia is defined as elevated blood lipid levels, and it is a significant public health issue globally and in Taiwan [1]. The World Health Organization (WHO) reported that approximately 39% of adults aged 25 and older have hyperlipidemia, and that this is associated with increased risks of cardiovascular diseases such as heart disease and stroke—leading causes of death worldwide [2]. In Taiwan, around 44% of adults aged 20 and above have hyperlipidemia, and this has been shown to significantly contribute to the incidence and mortality of cardiovascular disease, the second leading cause of death in Taiwan after cancer [3]. Hyperlipidemia is strongly linked to modifiable lifestyle factors such as a diet rich in saturated or trans fats, physical inactivity, smoking, and obesity, with additional influences including underlying medical conditions and certain medications that can exacerbate elevated lipid levels [4]. The prevalence of these risk factors continues to rise, placing a substantial burden on public health systems, increasing individual healthcare costs, and impacting quality of life. Therefore, enhancing prevention and management strategies for hyperlipidemia and promoting healthy lifestyles are essential for reducing cardiovascular disease incidence [5]. Protective measures include adopting a balanced diet (e.g., the Mediterranean diet), engaging in regular aerobic exercise, maintaining a healthy weight, and using lipid‐lowering medications such as statins [6]. In addition, stopping smoking and moderating alcohol consumption can improve lipid profiles and reduce cardiovascular risk [7]. Effective management of risk and protective factors such as reducing serum lipids is crucial for reducing the prevalence and impact of hyperlipidemia [8].

Hyperuricemia is defined as an elevated serum uric acid (SUA) level, and it adversely affects metabolic health by contributing to endothelial dysfunction, oxidative stress, and systemic inflammation. Moreover, it has also been associated with metabolic syndrome [9]. Uric acid is an end product derived from purine nucleotides through various metabolic pathways, and it is excreted by the urine (70%) and digestive (30%) systems [10]. The prevalence of hyperuricemia is rising globally and in Taiwan, where it affects 30.4% of the population and continues to increase annually [11]. The relationship between hyperuricemia and hyperlipidemia is particularly concerning, as high uric acid levels are associated with dyslipidemia, characterized by elevated triglycerides, increased low‐density lipoprotein cholesterol (LDL‐C), and reduced high‐density lipoprotein cholesterol (HDL‐C) [12]. This interplay increases the risk of atherosclerosis and cardiovascular disease, underscoring the importance of managing these interconnected conditions to prevent extensive metabolic and cardiovascular complications [13]. Previous studies have shown that hyperuricemia is closely associated with hypertriglyceridemia, with elevated lipoprotein lipase potentially contributing to higher levels of free fatty acids and reduced uric acid clearance, and also that hyperuricemia is associated with obesity and insulin resistance [14]. Despite the well‐documented impact of hyperuricemia on metabolic health and its association with cardiovascular and renal conditions, its specific relationship with hyperlipidemia is unclear, with research across different racial and ethnic groups currently ongoing [15].

This study aimed to investigate the association between elevated levels of uric acid and hyperlipidemia in a large‐scale, population‐based cohort in Taiwan, providing critical insights into their combined impact on metabolic health.

## 2 Materials and Methods

### 2.1 Study Design and Study Population

In response to the demographic shift towards an aging population in Taiwan, the Ministry of Health and Welfare established the Taiwan Biobank (TWB) with the goal of preventing chronic diseases and promoting healthcare. The TWB cohort comprises participants aged 30 to 70 years who have not received a previous diagnosis of cancer. The TWB database encompasses comprehensive data including medical, genetic, and lifestyle factors, as documented in previous studies [16, 17]. Ethical approval for the TWB was obtained from the Ethics and Governance Council of the TWB and the Institutional Review Board on Biomedical Science Research, Academia Sinica, Taiwan.

Participants in the TWB undergo a comprehensive enrollment process, during which they provide information on age and personal medical history, including hypertension and diabetes mellitus. A physical examination is conducted to gather data on height and weight, and the body mass index (BMI) is calculated (kg/m^2^). Fasting blood sampling is carried out to obtain data on biomarkers including total cholesterol, triglycerides, glucose, hemoglobin, triglycerides, total cholesterol, HDL‐C, LDL‐C, estimated glomerular filtration rate (eGFR) (calculated using the 4‐variable Modification of Diet in Renal Disease study equation [186 × serum creatinine −1.154 × age −0.203 × 0.742 (if female) × 1.212 (if Black)] [18]), and uric acid.

**FIGURE 1:**
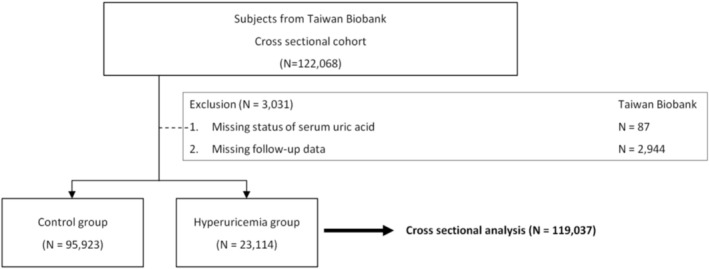
Study participants classified by the presence of hyperuricemia.

For the current investigation, 119,037 participants with available follow‐up data from the TWB were identified, excluding those with pre‐existing hyperlipidemia, those lacking basic information, and those missing uric acid data. The median follow‐up period was 4 years. All included participants provided written informed consent (Figure 1).

### 2.2 Ethics Statement

This study was approved by the Institutional Review Board of Kaohsiung Medical University Hospital (KMUHIRB‐E(I)‐20210058). All participants provided written informed consent, and the study adhered to the principles of the Declaration of Helsinki.

### 2.3 Definitions of Hyperuricemia and Hyperlipidemia

Hyperuricemia was defined as an SUA level > 7.0 mg/dL in men and 6.0 mg/dL in women. Hyperlipidemia was classified as a total cholesterol level ≥ 240 mg/dL, triglyceride level ≥ 200 mg/dL, or if the participants were taking lipid‐lowering medications. Those who did not meet these criteria were classified into the control group.

### 2.4 Covariates

A directed acyclic graph was used to identify potential confounders in the relationship between hyperuricemia and hyperlipidemia, including age, sex, smoking, drinking alcohol, obesity, diet, physical activity, hypertension, diabetes mellitus, dyslipidemia, and chronic kidney disease [19]. These covariates were recorded from self‐reported questionnaires, physical examinations, and blood tests.

### 2.5 Statistical Analysis

Participants were categorized into two groups: those with hyperuricemia and those without (control group). Continuous variables were summarized as means ± standard deviation (SD), while categorical variables were presented as percentages. To compare the two groups, independent *t*‐tests were employed for continuous variables, and chi‐square tests were used for categorical variables. The relationship between hyperuricemia and hyperlipidemia was assessed using logistic regression analysis, while multivariable linear regression was utilized to evaluate the association between SUA levels and lipid profiles. Statistical analyses were performed using R software (version 3.6.2, R Foundation for Statistical Computing, Vienna, Austria) and SPSS software (version 29.0, IBM Corp., Armonk, NY, USA). A *p*‐value of less than 0.05 was deemed statistically significant. The authors confirmed that no Artificial Intelligence Generated Content (AIGC) tools, such as ChatGPT or other large language models, were used in the preparation of this manuscript to obey Wiley's policy.

## 3 Results

### 3.1 Basic Characteristics of the Study Population

In this study of 119,037 participants, hyperuricemia was associated with a more adverse cardiometabolic profile. The participants with hyperuricemia had a significantly higher BMI (26.50 ± 3.94 vs. 23.67 ± 3.54 kg/m^2^, *p* < 0.001), greater prevalence of smoking (37.6% vs. 24.7%, *p* < 0.001), alcohol consumption (13.9% vs. 7.2%, *p* < 0.001), hypertension (20.9% vs. 10.2%, *p* < 0.001), and diabetes (6.3% vs. 4.9%, *p* < 0.001). They also had worse lipid profiles, including higher levels of total cholesterol (201.75 ± 37.46 vs. 194.21 ± 35.26 mg/dL, *p* < 0.001), higher triglycerides (157.65 ± 127.12 vs. 105.35 ± 80.91 mg/dL, *p* < 0.001) and higher LDL‐C (127.59 ± 33.09 vs. 119.36 ± 31.20 mg/dL, *p* < 0.001), and lower HDL‐C (48.39 ± 11.54 vs. 56.05 ± 13.45 mg/dL, *p* < 0.001). In addition, they had a lower eGFR and higher fasting glucose and hemoglobin A1c levels. These findings underscore the significant cardiometabolic risks linked to elevated SUA levels, highlighting the importance of comprehensive risk‐factor management in this population (Table 1).

**TABLE 1:** Baseline characteristics of the study subjects.

Characteristics	All	Hyperuricemia (−)	Hyperuricemia (+)	*p*
	*N* = 119,037	*N* = 95,923	*N* = 23,114	
Age (year)	49.89 ± 10.95	49.68 ± 10.88	50.76 ± 11.18	< 0.001
BMI (kg/m^2^)	24.22 ± 3.79	23.67 ± 3.54	26.50 ± 3.94	< 0.001
Male, *n* (%)	42,724 (35.9)	29,984 (35.2)	12,740 (29.8)	< 0.001
Ever smoking, *n* (%)	32,438 (27.3)	23,738 (24.7)	8700 (37.6)	< 0.001
Alcohol status, ever, *n* (%)	10,139 (8.5)	6922 (7.2)	3217 (13.9)	< 0.001
Exercise habits, yes, *n* (%)	48,351 (40.6)	38,680 (40.3)	9671 (41.8)	< 0.001
Ever‐married, yes, *n* (%)	102,873 (86.4)	82,706 (86.2)	20,167 (87.3)	< 0.001
Systolic BP, mmHg	120.52 ± 18.67	118.93 ± 18.36	127.15 ± 18.46	0.535
Diastolic BP, mmHg	73.83 ± 11.40	72.74 ± 11.11	78.38 ± 11.47	< 0.001
*Comorbidities*				
Hypertension, *n* (%)	14,596 (12.3)	9759 (10.2)	4837 (20.9)	< 0.001
Diabetes, *n* (%)	6128 (5.1)	4664 (4.9)	1464 (6.3)	< 0.001
Hyperlipidemia, *n* (%)	8880 (7.5)	6376 (6.6)	2504 (10.8)	< 0.001
*Lab data*				
eGFR (mL/min/1.73 m^2^)	103.31 ± 23.88	106.32 ± 23.51	90.84 ± 21.22	< 0.001
Hemoglobin (g/dL)	13.75 ± 1.60	13.60 ± 1.59	14.38 ± 1.49	< 0.001
Albumin (g/dL)	4.52 ± 0.23	4.51 ± 0.23	4.57 ± 0.25	< 0.001
Fasting glucose (mg/dL)	95.90 ± 20.67	95.29 ± 21.07	98.43 ± 18.74	0.002
Hemoglobin A1c (%)	5.76 ± 0.80	5.74 ± 0.81	5.86 ± 0.74	0.006
Total cholesterol (mg/dL)	195.67 ± 35.82	194.21 ± 35.26	201.75 ± 37.46	< 0.001
Triglyceride (mg/dL)	115.50 ± 94.02	105.35 ± 80.91	157.65 ± 127.12	< 0.001
HDL cholesterol (mg/dL)	54.56 ± 13.45	56.05 ± 13.45	48.39 ± 11.54	< 0.001
LDL cholesterol (mg/dL)	120.96 ± 31.74	119.36 ± 31.20	127.59 ± 33.09	< 0.001
Serum uric acid (mg/dL)	5.43 ± 1.43	4.93 ± 0.99	7.50 ± 1.02	< 0.001

*Note:* Reference ranges: Total cholesterol < 240 mg/dL, Triglyceride < 200 mg/dL, LDL‐C < 130 mg/dL, HDL‐C > 40 mg/dL in men and > 50 mg/dL in women, Serum uric acid > 7.0 mg/dL in men and 6.0 mg/dL in women.

### 3.2 Association Between the Presence of Hyperlipidemia and Hyperuricemia

In univariate analysis, hyperuricemia was significantly associated with hyperlipidemia, with an odds ratio (OR) of 1.706 (95% CI: 1.625–1.791, *p* < 0.001) (Table 2). This association was robust even when considering other factors such as age, BMI, sex, smoking status, alcohol consumption, and blood pressure, all of which also showed significant correlations with hyperlipidemia (Table 2).

**TABLE 2:** Univariate binary logistic regression analysis between parameters and hyperlipidemia.

Parameters	Odds ratio (95% CI)	*p*
Age (per 1 year)	1.078 (1.075 to 1.081)	< 0.001
BMI (per 1 kg/m^2^)	1.097 (1.092 to 1.103)	< 0.001
Male, vs. female	1.491 (1.428 to 1.558)	< 0.001
Smoking status, ever (vs. never)	1.435 (1.371 to 1.503)	< 0.001
Alcohol status, ever (vs. never)	1.558 (1.457 to 1.666)	< 0.001
Exercise habits, yes (vs. no)	1.555 (1.489 to 1.624)	< 0.001
Ever‐married, yes (vs. no)	2.376 (2.180 to 2.590)	< 0.001
Systolic blood pressure (per 1 mmHg)	1.022 (1.021 to 1.023)	< 0.001
Diastolic blood pressure (per 1 mmHg)	1.023 (1.021 to 1.024)	< 0.001
Hypertension, yes (vs. no)	6.372 (6.082 to 6.676)	< 0.001
Diabetes, yes (vs. no)	6.952 (6.553 to 7.376)	< 0.001
eGFR (per 1 mL/min/1.73 m^2^)	0.983 (0.982 to 0.984)	< 0.001
Hemoglobin (per 1 g/dL)	1.164 (1.148 to 1.181)	< 0.001
Albumin (per 1 g/dL)	1.763 (1.605 to 1.936)	< 0.001
Fasting glucose (per 1 mg/dL)	1.013 (1.013 to 1.014)	< 0.001
Hemoglobin A1c (per 1%)	1.536 (1.509 to 1.565)	< 0.001
Hyperuricemia, yes (vs. no)	1.706 (1.625 to 1.791)	< 0.001

After adjusting for potential confounders in multivariate analysis, hyperuricemia remained significantly associated with hyperlipidemia (OR: 1.102, 95% CI: 1.041–1.165, *p* < 0.001) (Table 3). These results confirmed that the relationship between hyperuricemia and hyperlipidemia was independent of other risk factors including age, BMI, hypertension, and diabetes (Table 3). In addition, the results underscore the persistent and independent role of elevated uric acid levels in the development of hyperlipidemia, highlighting its potential importance in comprehensive metabolic risk assessments.

**TABLE 3:** Multivariate binary logistic regression analysis between parameters and hyperlipidemia.

Parameters	Odds ratio (95% CI)	*p*
Age (per 1 year)	1.062 (1.059 to 1.066)	< 0.001
BMI (per 1 kg/m^2^)	1.058 (1.051 to 1.065)	< 0.001
Male, vs. female	0.865 (0.807 to 0.927)	< 0.001
Smoking status, ever (vs. never)	1.216 (1.143 to 1.294)	< 0.001
Alcohol status, ever (vs. never)	1.111 (1.027 to 1.201)	0.009
Exercise habits, yes (vs. no)	1.013 (0.965 to 1.063)	0.612
Ever‐married, yes (vs. no)	1.028 (0.936 to 1.130)	0.560
Systolic blood pressure (per 1 mmHg)	0.996 (0.994 to 0.997)	< 0.001
Diastolic blood pressure (per 1 mmHg)	0.998 (0.995 to 1.001)	0.192
Hypertension, yes (vs. no)	3.221 (3.049 to 3.403)	< 0.001
Diabetes, yes (vs. no)	2.803 (2.591 to 3.032)	< 0.001
eGFR (per 1 mL/min/1.73 m^2^)	0.997 (0.996 to 0.998)	< 0.001
Hemoglobin (per 1 g/dL)	1.058 (1.037 to 1.080)	< 0.001
Albumin (per 1 g/dL)	2.719 (2.439 to 3.031)	< 0.001
Fasting glucose (per 1 mg/dL)	0.996 (0.995 to 0.997)	< 0.001
Hemoglobin A1c (per 1%)	1.203 (1.156 to 1.241)	< 0.001
Hyperuricemia, yes (vs. no)	1.102 (1.041 to 1.165)	< 0.001

### 3.3 Association Between the Presence of Hyperuricemia and Hyperlipidemia in Participants Stratified by Age and Sex

In unadjusted models, hyperuricemia was associated with a higher odds of hyperlipidemia across all subgroups. However, after adjusting for confounders, the association remained significant in younger individuals; specifically, females aged ≤ 65 years with hyperuricemia (adjusted OR: 1.124, 95% CI: 1.027–1.230, *p* = 0.011), while males aged ≤ 65 years (adjusted OR: 1.098, 95% CI: 1.007–1.198, *p* = 0.035). Conversely, no significant association was observed in those aged > 65 years after adjustment, regardless of sex (Table 4). These findings suggest that the link between hyperuricemia and hyperlipidemia may be stronger in younger individuals, highlighting the need for targeted interventions and further research to understand potential age‐ and sex‐related mechanisms.

**TABLE 4:** Univariate and Multivariable Binary Logistic Analyses for the Prevalence of Hyperlipidemia in Subgroup Analyses.

Characteristics	Crude odds ratio (95% CI)	*p*	Adjusted odds ratio (95% CI)	*p*
Sex, female, > 65 years old (*N* = 6453)				
Hyperuricemia (+)	1.268 (1.083 to 1.484)	0.003	0.933 (0.781 to 1.114)	0.442
Hyperuricemia (−)	1.00			
Sex, female, ≤ 65 years old (*N* = 69,860)				
Hyperuricemia (+)	2.192 (2.028 to 2.329)	< 0.001	1.124 (1.027 to 1.230)	0.011
Hyperuricemia (−)	1.00			
Sex, male, > 65 years old (*N* = 4975)				
Hyperuricemia (+)	1.326 (1.122 to 1.567)	< 0.001	0.959 (0.796 to 1.155)	0.658
Hyperuricemia (−)	1.00			
Sex, male, ≤ 65 years old (*N* = 37,749)				
Hyperuricemia (+)	1234 (1.143 to 1.332)	< 0.001	1.098 (1.007 to 1.198)	0.035
Hyperuricemia (−)	1.00			

*Note:* Multivariable model: adjustment for age, sex, body mass index, smoking status, alcohol status, exercise habits, ever‐married, systolic blood pressure, diastolic blood pressure, history of hypertension, history of diabetes mellitus, estimated Glomerular filtration rate, hemoglobin, albumin, fasting glucose, hemoglobin A1c.

Abbreviation: CI: confidence interval.

### 3.4 Dose–Response Effect Between SUA and the Risk of Hyperlipidemia

To investigate the dose–response effect between SUA and the risk of hyperlipidemia, we divided the individuals aged < 65 years (younger population) by their SUA levels. Using SUA ≤ 4.0 mg/dL as the reference, no significant increase in the prevalence of hyperlipidemia was observed in those with an SUA level of 4.0–5.0 mg/dL (adjusted OR: 0.929, 95% CI: 0.846–1.022, *p* = 0.129), 5.0–6.0 mg/dL (adjusted OR: 1.000, 95% CI: 0.910–1.100, *p* = 0.995), 6.0–7.0 mg/dL (adjusted OR: 0.953, 95% CI: 0.858–1.059, *p* = 0.370), or 7.0–8.0 mg/dL (adjusted OR: 1.086, 95% CI: 0.963–1.224, *p* = 0.177). However, those with an SUA level > 8.0 mg/dL were significantly associated with an increased risk of hyperlipidemia (adjusted OR: 1.194, 95% CI: 1.041–1.369, *p* = 0.011) (Table 5). These findings indicate that while mildly elevated SUA levels may not independently predict hyperlipidemia in younger individuals, significantly elevated levels (> 8.0 mg/dL) are associated with a higher risk.

**TABLE 5:** Odds ratio for the prevalence of hyperlipidemia according to the level of serum uric acid in younger individuals (*N* = 107,609).

Characteristics	Number of cases (%)	Number at risk	Adjusted odds ratio (95% CI)	*p*
Serum uric acid ≤ 4.0 mg/dL	16.6	17,904	1.00 (reference)	
4.0 mg/dL < serum uric acid ≤ 5.0 mg/dL	28.3	30,495	0.929 (0.846 to 1.022)	0.129
5.0 mg/dL < serum uric acid ≤ 6.0 mg/dL	25.7	27,615	1.000 (0.910 to 1.100)	0.995
6.0 mg/dL < serum uric acid ≤ 7.0 mg/dL	16.4	17,641	0.953 (0.858 to 1.059)	0.370
7.0 mg/dL < serum uric acid ≤ 8.0 mg/dL	8.3	8898	1.086 (0.963 to 1.224)	0.177
Serum uric acid > 8.0 mg/dL	4.7	5056	1.194 (1.041 to 1.369)	0.011

## 4 Discussion

Our findings showed a significant and independent association between hyperuricemia and hyperlipidemia, emphasizing the link between a detrimental cardiometabolic profile and elevated SUA levels. Considering the large cohort of 119,037 Taiwanese participants, our findings provide comprehensive evidence that hyperuricemia should be considered a key factor in hyperlipidemia risk assessments. This study is one of the most extensive analyses in Asia to thoroughly investigate the relationship between SUA levels and lipid disorders.

Our finding of the significant association between hyperuricemia and hyperlipidemia is consistent with other studies. Studies from Western countries, such as the National Health and Nutrition Examination Survey (NHANES) in the United States, have highlighted the association between hyperuricemia and metabolic syndrome components including hypertension and dyslipidemia. These studies found that hyperuricemia was not only correlated with increased cardiovascular risk, but that it may also act as an independent marker of metabolic disturbances [20]. Our study reinforces these findings, showing a significant independent relationship between hyperuricemia and hyperlipidemia in a Taiwanese cohort (adjusted OR = 1.102, 95% CI: 1.041–1.165, *p* < 0.001). This consistency across diverse populations underscores the universality of the impact of hyperuricemia on lipid metabolism, and supports its role as a critical factor in cardiometabolic health.

Our findings are also consistent with studies in Asia that have examined the association between hyperuricemia and lipid disorders. For example, research in China and Korea has reported a significant association between elevated SUA levels and dyslipidemia, and that individuals with higher uric acid levels had an increased odds of adverse lipid profiles including elevated triglycerides and LDL‐C, along with lower HDL‐C [21, 22]. In contrast, some studies have reported no significant association between SUA levels and lipid disorders. For example, a cross‐sectional study conducted in Iran with 140 patients found no statistically significant correlation between SUA levels and the extent of atherosclerosis [23]. The discrepancy between the results of our study and other studies could be due to differences in study design, sample size, and populations. Our large sample size and comprehensive multivariable adjustments provide a more robust analysis, allowing us to draw stronger conclusions about the independent role of hyperuricemia in the development of hyperlipidemia.

A key finding of our study is the significant age and sex differences in the association between hyperuricemia and hyperlipidemia. The link was stronger in younger women (adjusted OR: 1.124, 95% CI: 1.027–1.230, *p* = 0.011) than in younger men (adjusted OR: 1.098, 95% CI: 1.007–1.198, *p* = 0.035), with no significant association in individuals over 65 years (all *p* > 0.05). This is consistent with recent large‐scale data showing that the excess cardiovascular risk from gout is greatest in younger individuals (< 45 years) and women [24]. Previous studies demonstrated that elevated SUA levels were a predictor of metabolic disturbances including prediabetes and insulin resistance, particularly in younger adults, suggesting that hormonal and physiological factors may influence these associations [25]. Taken together, these findings suggest that early‐onset hyperuricemia may confer a higher cardiometabolic risk and underscore the importance of tailored prevention strategies based on both age and sex.

Beyond epidemiological associations, emerging evidence from multi‐omics analyses highlights a mechanistic link between hyperuricemia, gout, and lipid metabolism. Early‐onset hyperuricemia and gout are associated with distinct plasma lipid signatures, including the upregulation of phosphatidylethanolamines (PEs) and downregulation of lysophosphatidylcholine (LPC) plasmalogens, changes that are partially corrected by urate‐lowering therapy [26, 27].

The mechanisms behind the observed associations likely involve oxidative stress, endothelial dysfunction, and systemic inflammation induced by hyperuricemia [28]. At the molecular level, studies further demonstrate that hyperuricemia induces oxidative stress, endothelial dysfunction, and systemic inflammation, with hepatic LPCAT3 and CXCL‐13 upregulation driving lipid disturbances, characterized by elevated triglycerides and LDL‐C and reduced HDL‐C [29, 30]. These processes are mediated through inflammatory cytokine activation, such as IL‐6, TNF‐α, TGF‐β1, and mitochondrial dysfunction, reinforcing the causal role of uric acid in dyslipidemia and cardiovascular disease [31, 32].

At the cellular level, high SUA impairs endothelial cell function and bile acid synthesis via p38MAPK/NF‐κB signaling, leading to lipid abnormalities [33]. Animal and human lipidomic studies consistently implicate glycerophospholipid metabolism as a central pathway affected by hyperuricemia, with specific lipid biomarkers differentiating asymptomatic hyperuricemia, gout, and healthy controls [27, 34]. These mechanistic insights complement our epidemiological findings, suggesting that managing SUA may not only reduce gout flares but also correct underlying lipid metabolic derangements and thereby lower cardiovascular risk.

Furthermore, large‐scale clinical data confirm that gout confers an independent excess risk for multiple cardiovascular diseases beyond atherosclerosis, including heart failure, arrhythmias, valve disease, and thromboembolic events, with risk particularly elevated in younger individuals and women [24]. Importantly, previous studies have revealed higher cardiometabolic risk scores in patients with hyperuricemia and gout, correlating with inflammatory markers and lipid atherogenicity, suggesting potential utility in early cardiovascular risk identification [35].

This study has several limitations that should be acknowledged. First, the cross‐sectional nature of the research limits causal inferences between hyperuricemia and hyperlipidemia. Longitudinal studies are needed to confirm our findings. Second, the relatively small size of the control cohort compared with the hyperuricemia group may reduce statistical power and generalizability. Nevertheless, the large overall sample, comprehensive adjustments, and consistent subgroup findings strengthen the robustness of our conclusions. In addition, while our sample size was large and the adjustments for confounders were comprehensive, residual confounding cannot be entirely ruled out. Despite these limitations, our findings provide robust evidence for the independent role of hyperuricemia in hyperlipidemia and underscore the need for further research to explore the long‐term effects of uric acid management on lipid profiles and cardiovascular health.

## 5 Conclusion

This large‐scale population‐based study conducted in Taiwan provides compelling evidence of a significant independent association between hyperuricemia and hyperlipidemia, reinforcing the role of elevated SUA as a marker of adverse lipid profiles. Our findings suggest that SUA levels should be considered in routine metabolic risk assessments to better identify individuals at increased cardiovascular risk. The observed sex‐ and age‐specific differences further underscore the need for tailored clinical approaches. Comprehensive management strategies addressing both hyperuricemia and hyperlipidemia could play a crucial role in reducing cardiovascular risk. Further research is warranted to elucidate the underlying pathophysiological mechanisms linking these conditions and evaluate the effectiveness of interventions targeting hyperuricemia to alleviate its impact on lipid metabolism and overall cardiovascular health.

## Funding

The Ministry of Science and Technology research grant in Taiwan (MOST 111‐2314‐B‐037‐061; MOST 112‐2314‐B‐037‐115‐MY2; NSTC 111‐2218‐E‐037‐001; NSTC 112‐2218‐E‐037‐001; NSTC113‐2218‐E‐037‐001; NSTC 112‐2314‐B‐037‐127; NSTC 113‐2314‐B‐037‐016; NSTC114‐2314‐B‐037‐024; NSTC114‐2314‐B‐037‐025); the Kaohsiung Medical University Research Center Grant (KMU‐TC109A01‐1; NHRIKMU‐113‐I001; KMUH112‐2R59; KMUH113‐3R52); and Kaohsiung Municipal Siaogang Hospital (S‐111‐16; kmhk‐112‐23; S‐112‐01; S‐113‐01; I‐113‐01; H‐113‐10).

## Data Availability

The data that support the findings of this study are available from Taiwan Biobank but restrictions apply to the availability of these data, which were used under license for the current study, and so are not publicly available. Data are however available from the authors upon reasonable request and with permission of Taiwan Biobank. Please send requests for data access to Jiun‐Hung Geng, MD, Department of Urology, Kaohsiung Medical University Hospital, Kaohsiung Medical University.

## References

[1] P.‐H. Huang , Y.‐W. Lu , Y.‐L. Tsai , et al., “2022 Taiwan Lipid Guidelines for Primary Prevention,” Journal of the Formosan Medical Association 121, no. 12 (2022): 2393–2407, 10.1016/j.jfma.2022.05.010. 35715290

[2] J. J. N. Noubiap , J. R. N. Nansseu , J. J. R. Bigna , A. M. Jingi , and A. P. Kengne , “Prevalence and Incidence of Dyslipidaemia Among Adults in Africa: A Systematic Review and Meta‐Analysis Protocol,” BMJ Open 5, no. 3 (2015): e7404, 10.1136/bmjopen-2014-007404. PMC4368904 25783427

[3] C.‐F. Tsao , C.‐M. Chang , S.‐W. Weng , P.‐W. Wang , C.‐Y. Lin , and S.‐N. Lu , “Identifying Endemic Areas and Estimating the Prevalence of Hyperlipidemia in Taiwan's Townships,” Journal of the Formosan Medical Association 120, no. 1 (2021): 460–465, 10.1016/j.jfma.2020.05.031. 32631706

[4] S. Karr , “Epidemiology and Management of Hyperlipidemia,” American Journal of Managed Care 23, no. 9 (2017): S139–S148. 28978219

[5] T. A. Pearson , “Understanding the Impact of Hyperlipidemia Treatment on Medical Expenditures for Cardiovascular Disease,” Medical Care 55, no. 1 (2017): 1–3, 10.1097/mlr.0000000000000682. 27930502

[6] J. Delgado‐Lista , P. Perez‐Martinez , A. Garcia‐Rios , A. I. Perez‐Caballero , F. Perez‐Jimenez , and J. Lopez‐Miranda , “Mediterranean Diet and Cardiovascular Risk: Beyond Traditional Risk Factors,” Critical Reviews in Food Science and Nutrition 56, no. 5 (2014): 788–801, 10.1080/10408398.2012.726660. 25118147

[7] D. P. Mikhailidis , J. A. Papadakis , and E. S. Ganotakis , “Smoking, Diabetes and Hyperlipidaemia,” Journal of the Royal Society of Health 118, no. 2 (1998): 91–93, 10.1177/146642409811800209. 10076642

[8] Y. S. Yao , T. D. Li , and Z. H. Zeng , “Mechanisms Underlying Direct Actions of Hyperlipidemia on Myocardium: An Updated Review,” Lipids in Health and Disease 19, no. 1 (2020): 23, 10.1186/s12944-019-1171-8. 32035485 PMC7007679

[9] C. Ben Salem , R. Slim , N. Fathallah , and H. Hmouda , “Drug‐Induced Hyperuricaemia and Gout,” Rheumatology 56, no. 5 (2017): 679–688, 10.1093/rheumatology/kew293. 27498351

[10] W. G. Lima , M. E. Martins‐Santos , and V. E. Chaves , “Uric Acid as a Modulator of Glucose and Lipid Metabolism,” Biochimie 116 (2015): 17–23, 10.1016/j.biochi.2015.06.025. 26133655

[11] Q. Yu , “Prevalence and Metabolic Factors of Hyperuricemia in an Elderly Agricultural and Fishing Population in Taiwan,” Archives of Rheumatology 32, no. 2 (2017): 149–157, 10.5606/ArchRheumatol.2017.6075. 30375557 PMC6190989

[12] S. Chen , X. Guo , S. Dong , et al., “Association Between the Hypertriglyceridemic Waist Phenotype and Hyperuricemia: A Cross‐Sectional Study,” Clinical Rheumatology 36, no. 5 (2017): 1111–1119, 10.1007/s10067-017-3559-z. 28185015

[13] K. Li , K. Li , Q. Yao , et al., “The Potential Relationship of Coronary Artery Disease and Hyperuricemia: A Cardiometabolic Risk Factor,” Heliyon 9, no. 5 (2023): e16097, 10.1016/j.heliyon.2023.e16097. 37215840 PMC10199191

[14] H. Nakamura , “Association of Hyperuricemia With Hyperlipidemia and Obesity,” Nihon Rinsho 54, no. 12 (1996): 3289–3292. 8976107

[15] Y. Zhang , M. Zhang , X. Yu , et al., “Association of Hypertension and Hypertriglyceridemia on Incident Hyperuricemia: An 8‐Year Prospective Cohort Study,” Journal of Translational Medicine 18, no. 1 (2020): 409, 10.1186/s12967-020-02590-8. 33129322 PMC7603698

[16] C.‐H. Chen , J.‐H. Yang , C. W. K. Chiang , et al., “Population Structure of Han Chinese in the Modern Taiwanese Population Based on 10,000 Participants in the Taiwan Biobank Project,” Human Molecular Genetics 25, no. 24 (2016): 5321–5331, 10.1093/hmg/ddw346. 27798100 PMC6078601

[17] C. T. Fan , T. H. Hung , and C. K. Yeh , “Taiwan Regulation of Biobanks,” Journal of Law, Medicine & Ethics 43, no. 4 (2015): 816–826, 10.1111/jlme.12322. 26711420

[18] A. S. Levey , J. P. Bosch , J. B. Lewis , T. Greene , N. Rogers , and D. Roth , “A More Accurate Method to Estimate Glomerular Filtration Rate From Serum Creatinine: A New Prediction Equation. Modification of Diet in Renal Disease Study Group,” Annals of Internal Medicine 130, no. 6 (1999): 461–470, 10.7326/0003-4819-130-6-199903160-00002. 10075613

[19] J. Stewart , T. McCallin , J. Martinez , S. Chacko , and S. Yusuf , “Hyperlipidemia,” Pediatrics in Review 41, no. 8 (2020): 393–402, 10.1542/pir.2019-0053. 32737252

[20] A. J. Luk and P. A. Simkin , “Epidemiology of Hyperuricemia and Gout,” American Journal of Managed Care 11, no. 15 (2005): S435–S442. 16300457

[21] S. Pang , Q. Jiang , P. Sun , et al., “Hyperuricemia Prevalence and Its Association With Metabolic Disorders: A Multicenter Retrospective Real‐World Study in China,” Annals of Translational Medicine 9, no. 20 (2021): 1550, 10.21037/atm-21-5052. 34790756 PMC8576711

[22] M. Son , J. Seo , and S. Yang , “Association Between Dyslipidemia and Serum Uric Acid Levels in Korean Adults: Korea National Health and Nutrition Examination Survey 2016–2017,” PLoS One 15, no. 2 (2020): e0228684, 10.1371/journal.pone.0228684. 32059030 PMC7021293

[23] F. Ashtarani , M. Farhadian , S. Keshtkar , and M. Bayanati , “Assessment of the Relationship Between Serum Uric Acid Level and Atherosclerosis Burden in Patients Undergoing Coronary Angiography in Ekbatan (Farshchian) Hospital, Hamadan 2015,” Artery Research 19 (2017): 38, 10.1016/j.artres.2017.06.002.

[24] L. D. Ferguson , G. Molenberghs , G. Verbeke , et al., “Gout and Incidence of 12 Cardiovascular Diseases: A Case‐Control Study Including 152 663 Individuals With Gout and 709 981 Matched Controls,” Lancet Rheumatol 6, no. 3 (2024): e156–e167, 10.1016/s2665-9913(23)00338-7. 38383089

[25] E. Krishnan , B. J. Pandya , L. Chung , A. Hariri , and O. Dabbous , “Hyperuricemia in Young Adults and Risk of Insulin Resistance, Prediabetes, and Diabetes: A 15‐Year Follow‐Up Study,” American Journal of Epidemiology 176, no. 2 (2012): 108–116, 10.1093/aje/kws002. 22753829

[26] A. Kvasnicka , D. Friedecky , R. Brumarova , et al., “Alterations in Lipidome Profiles Distinguish Early‐Onset Hyperuricemia, Gout, and the Effect of Urate‐Lowering Treatment,” Arthritis Research & Therapy 25, no. 1 (2023): 234, 10.1186/s13075-023-03204-6. 38042879 PMC10693150

[27] S. Liu , Y. Wang , H. Liu , et al., “Serum Lipidomics Reveals Distinct Metabolic Profiles for Asymptomatic Hyperuricemic and Gout Patients,” Rheumatology (Oxford) 61, no. 6 (2022): 2644–2651, 10.1093/rheumatology/keab743. 34599805

[28] Z. D. Wu , X. K. Yang , Y. S. He , et al., “Environmental Factors and Risk of Gout,” Environmental Research 212 (2022): 113377, 10.1016/j.envres.2022.113377. 35500858

[29] N. Liu , Q. Sun , H. Xu , et al., “Hyperuricemia Induces Lipid Disturbances Mediated by LPCAT3 Upregulation in the Liver,” FASEB Journal 34, no. 10 (2020): 13474–13493, 10.1096/fj.202000950R. 32780898

[30] J. Meng , Q. Lv , A. Sui , et al., “Hyperuricemia Induces Lipid Disturbances by Upregulating the CXCL‐13 Pathway,” American Journal of Physiology. Gastrointestinal and Liver Physiology 322, no. 2 (2022): G256–G267, 10.1152/ajpgi.00285.2021. 34935515

[31] X. Wei , M. Zhang , S. Huang , et al., “Hyperuricemia: A Key Contributor to Endothelial Dysfunction in Cardiovascular Diseases,” FASEB Journal 37, no. 7 (2023): e23012, 10.1096/fj.202300393R. 37272854

[32] H. Yanai , H. Adachi , M. Hakoshima , and H. Katsuyama , “Molecular Biological and Clinical Understanding of the Pathophysiology and Treatments of Hyperuricemia and Its Association With Metabolic Syndrome, Cardiovascular Diseases and Chronic Kidney Disease,” International Journal of Molecular Sciences 22, no. 17 (2021): 9221, 10.3390/ijms22179221. 34502127 PMC8431537

[33] W. Huang , M. Zhang , Q. Qiu , et al., “Metabolomics of Human Umbilical Vein Endothelial Cell‐Based Analysis of the Relationship Between Hyperuricemia and Dyslipidemia,” Nutrition, Metabolism, and Cardiovascular Diseases 34, no. 6 (2024): 1528–1537, 10.1016/j.numecd.2024.02.001. 38508990

[34] F. Yang , M. Liu , N. Qin , et al., “Lipidomics Coupled With Pathway Analysis Characterizes Serum Metabolic Changes in Response to Potassium Oxonate Induced Hyperuricemic Rats,” Lipids in Health and Disease 18, no. 1 (2019): 112, 10.1186/s12944-019-1054-z. 31077208 PMC6511199

[35] A. Kvasnicka , D. Friedecky , B. Pisklakova , J. Rozhon , K. Pavelka , and B. Stiburkova , “Ceramide‐Based Risk Score: A Novel Laboratory Tool for Cardiovascular Risk Stratification in Hyperuricemia and Gout,” Vascular Pharmacology 159 (2025): 107495, 10.1016/j.vph.2025.107495. 40239856

